# Annotations

**Published:** 1892-07-16

**Authors:** 


### Annotations.

Lord Sandhurst has issued invitations to each of the London hospitals and kindred charities, inviting them to a conference at Spencer House at the end of the present month. The object of the conference is to take into consideration the findings of the Lords' Committee on Metropoli tan Hospitals. The chief recommendation was the appo int ment of a Central Board, representative of all the hospitals, with the view of promoting the increased support of the well-established institutions, and the prevention of mushroom hospitals, which at present increase at the will and pleasure of any individual who has a purpose to serve. The issue of this invitation has given rise to a good deal of discussion in hospital circles, and has revealed a strong feeling in certain quarters against co-operation. In some directions there is an inclination not to send a representative at all, or merely to appoint some-one as a matter of courtesy to Lord Sandhurst. The adoption of either course would be, we believe, a mis-take, seeing that now the Lords' Report has been issued it is of the first importance to ascertain the opinions and views of the hospital managers thereon with as little delay as possible. Every hospital committee should, therefore, send a representative to the Con-ference with instructions to support or oppose such of the recommendations of the Lords' Committee as they may think well. If this step be taken, then Lord Sandhurst's action must result in good, because it will clear the air and enable him to ascertain what are the views of the hospital managers upon the points raised by the Lords' Committee which are now ripe for settle-ment. For these reasons we would venture to urge every Committee to send a duly instructed representa-tive who they know can put the views of the body he represents tersely but plainly before the Conference.

**The Hospital Conference.**

Beyond the failure of one method of treatment for consumption after another, survives the desire to try every new expedient in the breasts of those whose nearest and dearest are suffering from the dire disease. There is a certain confidence inspired when we feel that the methods we adopt are such as are also followed by those in high quarters. The Grand Duke George of Russia, the Czar's second son, is undergoing a "cure," which will appear even ultra-modern to those who cling to the warm climate theory which still has many followers. The Grand Duke became so ill during his tour in India that his return was necessary, and since then he has been in the hands of the doctors for pulmonary disease, and has been passing the winter at Abbas-Tuman, in the Caucasus, and there it is that the vigorous treat-ment, applied in his case, is being carried out. The walls of his apartments, we learn, are bare and un-papered, the furniture is of plain wood or cane, all upholstering and curtains being entirely abolished, and his mattress is of the thinnest descrip-tion. Throughout the winter the windows have been kept continuously open, and only a very moderate fire has been allowed. The attendants appear to have found the temperature they have been forced to submit to very far from comfortable, but the medical advisers seem confident that the low temperature is beneficial to their patient as tending to destroy the bacillus and prevent the formation of tubercle. They maintain that the progress of the disease has been arrested, and express hopes that, if the treatment is continued, that the Grand Duke will be restored to health in two years' time. Those who have watched the progress of consumption, even in advanced stages, must have observed the increased vigour of patients during really severe but dry cold in winter. In one case that came under our own observation the health of the sufferer became so much better during a winter of un-usual severity and dryness that hopes of the arrest of the disease were entertained. The patient grew rapidly worse on the weather becoming mild, and by June all was over. Davos Platz and similar resorts are rapidly becoming more popular for consumptives, as the belief in the Riviera and warmer climates is steadily decreasing. Nevertheless the efficacy of such rigid treatment as the Grand Duke George is under-going has still to be proved.

**A Royal Consumptive.**

The General Election has caused far too little notice to be taken of a matter of pressing import-ance to every family of Londoners. An edict has been issued by certain of the vestries forbidding dust-men to remove vegetable stuff and other garbage which is ordered to be burnt by the householder, no matter how small the grate or how frightful the smell. The excuse given by the vestry authorities is that in view of the threatened outbreak of cholera, the authorities have decided to take special precautions, of which this is one. In con-sequence of the protests of the householders, the vestry authorities seem to have grown a little afraid of en-forcing "this special precaution" as an indirect denial was given on enquiry at their office. The *Daily Tele-graph* has, however, ascertained that an order to burn all vegetable refuse, as it would be refused by the dust-men, was undoubtedly given. Until the advent of the County Council and the passing of a new Act regu-lating the health of London, such an instance of bumble-headedness need have caused no surprise. Having re-gard to the powers now vested in the County Council, of the boasted determination to enforce reform by the majority of the new members of that body, and to the circumstance that Dr. Shirley Murphy is the medical officer of health for London, we are at a loss to understand why our new masters should remain silent, as they appear to have done in regard to this pressing question, affecting, as it must do, the health and comfort of every householder. If the authorities, whoever they may be, remain passive, or if the dustmen refuse in any case to remove the vegetable refuse and other dangerous rubbish, we would counsel the house-holders to combine in their own defence, and so throw the responsibility for what happens on the authori-ties. The simplest way to do this will be to em-ploy a man to place all the objectionable matters in the street which the dustmen refuse to remove. Such a step would compel the authorities to promptly remove it, and so rid the householders and the whole neighbour-hood of what must prove an increasing danger to health. We cannot, however, believe that either the County Council or the vestry authorities will be mad enough to permit festering impurities of the nature complained of to remain for even twenty-four hours in the back yard of any metropolitan premises, or that they will add insult to injury by requesting house-holders to add to the evil by attempting to burn veget-able stuff in grates which are not only unsuited to the purpose, but which often have to cook the food of more than one family.

